# Viable *Mycobacterium avium* subsp. *paratuberculosis* Colonizes Peripheral Blood of Inflammatory Bowel Disease Patients

**DOI:** 10.3390/microorganisms11061520

**Published:** 2023-06-07

**Authors:** Maria Manuela Estevinho, José Cabeda, Mafalda Santiago, Elisabete Machado, Ricardo Silva, Mary Duro, Inês Pita, Rui Morais, Guilherme Macedo, Tim J. Bull, Fernando Magro, Amélia Sarmento

**Affiliations:** 1 Department of Gastroenterology, Vila Nova de Gaia/Espinho Hospital Center, 4434-502 Vila Nova de Gaia, Portugal; mmestevinho@gmail.com; 2 Department of Biomedicine, Unit of Pharmacology and Therapeutics, Faculty of Medicine, University of Porto, 4050-313 Porto, Portugal; mafaldasap@gmail.com (M.S.); fm@med.up.pt (F.M.); 3 FP-I3ID, Universidade Fernando Pessoa, 4200-150 Porto, Portugal; jcabeda@ufp.edu.pt (J.C.); emachado@ufp.edu.pt (E.M.); ricardos@ufp.edu.pt (R.S.); mduro@ufp.edu.pt (M.D.); 4 Escola Superior de Saúde Fernando Pessoa, 4200-253 Porto, Portugal; 5 Centro Interdisciplinar de Investigação Marinha e Ambiental (CIIMAR, CIMAR), 4450-208 Matosinhos, Portugal; 6 UCIBIO—Applied Molecular Biosciences Unit, Laboratory of Microbiology, Department of Biological Sciences, REQUIMTE, Faculty of Pharmacy, University of Porto, 4050-313 Porto, Portugal; 7 Faculdade de Ciências da Saúde, Universidade Fernando Pessoa, 4200-150 Porto, Portugal; 8 LAQV@REQUIMTE, Faculty of Pharmacy, University of Porto, 4050-313 Porto, Portugal; 9 Department of Gastroenterology, Entre Douro e Vouga Hospital Center, 4520-211 Santa Maria da Feira, Portugal; 10 Department of Gastroenterology, São João University Hospital Center, 4200-319 Porto, Portugal; 11 Institute of Infection and Immunity, St George’s University of London, London SW17 ORE, UK; tbull@sgul.ac.uk; 12 I3S, Instituto de Investigação e Inovação em Saúde, Universidade do Porto, 4200-150 Porto, Portugal

**Keywords:** Crohn’s disease, *Escherichia coli*, *Mycobacterium avium*, pathobionts, ulcerative colitis

## Abstract

Pathobionts, particularly *Mycobacterium avium* subsp. *paratuberculosis* (MAP) and *Escherichia coli* isolates with adherence/invasive ability (AIEC) have been associated with inflammatory bowel disease (IBD), particularly Crohn’s disease (CD). This study aimed to evaluate the frequency of viable MAP and AIEC in a cohort of IBD patients. As such, MAP and *E. coli* cultures were established from faecal and blood samples (with a total *n* = 62 for each) of patients with CD (*n* = 18), ulcerative colitis (UC, *n* = 15), or liver cirrhosis (*n* = 7), as well as from healthy controls (HC, *n* = 22). Presumptive positive cultures were tested by polymerase chain reaction (PCR), for a positive confirmation of MAP or *E. coli* identity. *E. coli*-confirmed isolates were then tested for AIEC identity using adherence and invasion assays in the epithelial cell line of Caco-2 and survival and replication assays in the macrophage cell line of J774. MAP sub-culture and genome sequencing were also performed. MAP was more frequently cultured from the blood and faecal samples of patients with CD and cirrhosis. *E. coli* presumptive colonies were isolated from the faecal samples of most individuals, in contrast to what was registered for the blood samples. Additionally, from the confirmed *E. coli* isolates, only three had an AIEC-like phenotype (i.e., one CD patient and two UC patients). This study confirmed the association between MAP and CD; however, it did not find a strong association between the presence of AIEC and CD. It may be hypothesized that the presence of viable MAP in the bloodstream of CD patients contributes to disease reactivation.

## 1. Introduction

Several studies have pointed out that inflammatory bowel disease (IBD) pathogenesis is a combination of an inherited susceptibility, environmental factors, and altered mucosal immune responses, yet the underlying mechanisms remain elusive [1,2]. In the last decade, an increased number of single nucleotide polymorphisms (SNPs), epigenetic traits, and environmental factors such as dietary habits and microbial colonization have been suggested to play a role in the development of IBD [3,4,5]. Microbes may contribute to inflammatory bowel disease (IBD) in various ways. In fact, the composition of gut microbiota appears to be a significant predisposing factor, as dysbiosis has been frequently observed in IBD patients, especially in Crohn’s disease (CD) [3,4,5,6]. Pathobionts, which are potentially pathological microorganisms that are normally non-harming symbionts, have also been suggested to play a role as triggers for the development and exacerbation of the disease. The two pathobionts most commonly associated with IBD, particularly CD, are *Mycobacterium avium* subsp. *paratuberculosis* (MAP) and *Escherichia coli* isolates that are capable of adhering to and invading intestinal epithelial cells (adherent-invasive *E. coli*—AIEC) [7,8,9,10,11,12,13,14,15]. In fact, both pathobionts and opportunistic pathogens may cause enteropathogen-induced disruptions, triggering or worsening immunopathogenesis through prolonged inflammation [16,17]. MAP colonization has been associated with the increased production of pro-inflammatory cytokines by infected macrophages [17,18] and AIEC has been shown to impair autophagy, which is a pivotal process implicated in intestinal homeostasis, microbial balance, and modulation of the immune response to enteric bacteria [19,20,21]. Pathobiont infection by MAP and AIEC may also modulate gut microbiota and contribute to dysbiosis in IBD patients [22,23].

In a previous study conducted by our group [24,25], we assessed the prevalence of MAP and *E. coli* in the peripheral blood of CD and ulcerative colitis (UC) patients belonging to different groups (with disease activity or who were in remission, with or without infliximab therapy). Since MAP culture from human samples is challenging due to paucibacillary colonization and dormancy [26], we used real-time PCR for microbial DNA detection. We compared the results obtained in patients with CD or UC with those of healthy controls (HC) and cirrhotic patients to evaluate the effect of increased intestinal permeability on bacterial DNA detection. Our findings revealed a high frequency of cirrhotic patients positive for both MAP and *E. coli* DNA, followed by CD patients. While these results suggested that an increased intestinal permeability may contribute to greater access of microbial DNA to the blood, CD patients in remission or with active disease showed comparable results, supporting a possible association between CD and the presence of MAP and *E. coli* DNA in the blood; however, since only bacterial DNA was detected, our previous study did not allow us to conclude about the presence of viable circulating bacteria. Furthermore, the AIEC identity of *E. coli* DNA-positive samples could not be confirmed, since this trait can only be determined using phenotypic adhesion and invasion assays [14].

Therefore, in this study we further explored our previous findings to address colonization by viable bacteria. We established in vitro MAP and *E. coli* cultures from the faeces and blood samples of IBD patients and controls, screening the cultures over time. To successfully culture MAP, we used a recently described culture process, involving the addition of supplements that stimulate MAP growth [27]. We further analysed the AIEC identity of cultured *E. coli*-positive samples, that had not been possible to confirm in our previous study. Investigation of the possible links between colonization by viable pathobionts and IBD development is relevant, allowing us to further understand the disease pathogenesis.

## 2. Materials and Methods

### 2.1. Participants and Sample Collection

Forty adult patients, diagnosed with IBD and liver cirrhosis, were prospectively recruited at a Portuguese tertiary hospital (Gastroenterology Department, Centro Hospitalar Universitário de São João, Porto, Portugal). IBD was characterized using the Montreal classification and the clinical activity was scored using the Harvey–Bradshaw index. Patients with cirrhosis were classified according to the Child–Pugh classification. Twenty-two healthy controls were recruited from the users of Dra. Matilde Sampaio Clinical Analyses Laboratory. The study protocol conformed to the ethical guidelines of the 1975 Declaration and ethical approval was granted. Informed consent was obtained in accordance with the institutional review board regulations. Participant information (e.g., age, gender, disease duration, disease classification, disease activity, and therapy) is included in Table 1.

**Figure 1:**
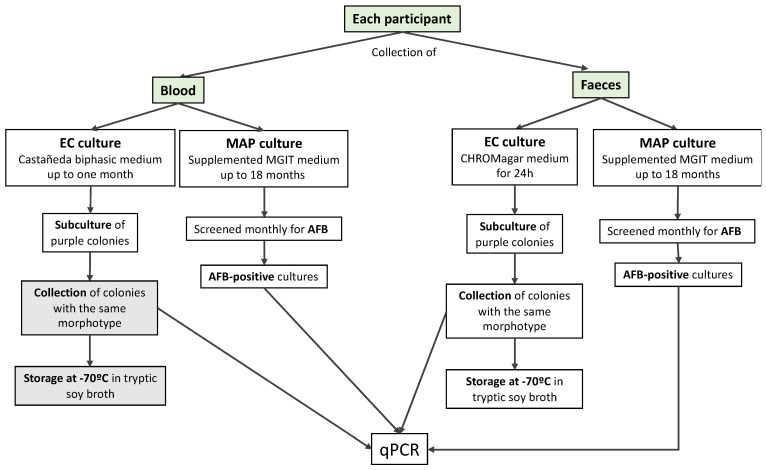
Flow chart of sample collection and processing; text boxes in grey refer to procedures not performed, due to a lack of positive colonies. Acid-fast bacilli (AFB), *Mycobacterium avium* subsp. *paratuberculosis* (MAP); *Escherichia coli* (EC).

**Figure 2:**
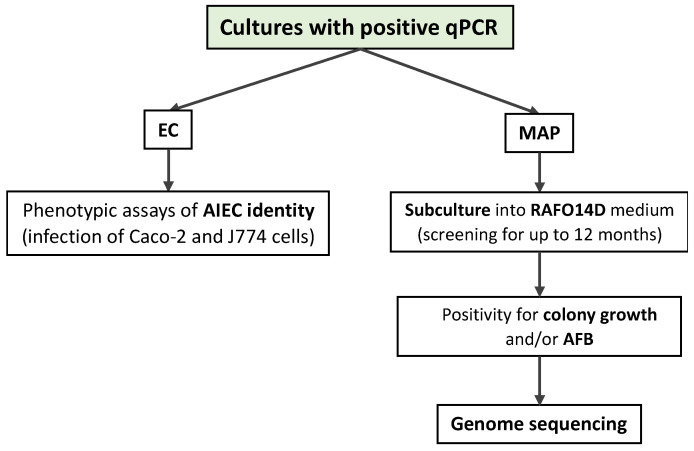
Processing of real-time PCR (qPCR)-positive samples.

The experimental protocol is summarized in Figure 1 and Figure 2.

Peripheral venous blood (10 mL) was collected from each participant, using citrate as an anticoagulant. Blood was divided into two aliquots (5 mL each) for either *E. coli* culture or MAP culture. Faeces were collected by each participant on the same or the previous day in appropriate provided, sterile containers and stored at 4 °C until analysis.

This study was conducted in agreement with the Declaration of Helsinki-Ethical principle for medical research involving human subjects and was approved by the local Ethics Committee (Comissão de Ética para a Saúde do Centro Hospitalar Universitário de São João, approval code 249/16, approval date 14 October 2016).

### 2.2. Blood Cultures

*E. coli* haemocultures were performed using a biphasic system for blood culture. Biphasic media, first developed for the isolation of Brucella, by Castañeda [28], consists of a liquid phase (broth) and an agar phase (slant) in the same bottle, in which the agar slant is inoculated by contacting with the broth. This allows the formation of colonies that can be easily observed and sub-cultured. For the preparation of the biphasic medium, 20 mL of Brain Heart Infusion (BHI, Liophilchem, Roseto degli Abruzzi, Italy)) agar was used as the slant, and a BHI broth as the liquid phase. Then, five mL of blood from each participant were inoculated in the liquid phase of the Castañeda system [28]. The flasks were shaken to allow the inoculation of the solid phase and were then incubated at 37 °C. For one month, the cultures were shaken weekly to allow contact between the phases and were screened daily for colony detection. The flasks were discarded when no colonies were detected after one month.

For the MAP haemocultures, 5 mL of blood from each participant was centrifuged for 10 min at 300× *g* and the supernatant fraction (the plasma) was discarded. The remaining cell pellet was diluted 1:10 in an ACK lysis buffer (Gibco, Thermo Fisher Scientific, Portugal) and incubated for 10 min at room temperature (the tubes were inverted 2–3 times during the incubation period). After incubation, the cell suspensions were centrifuged at 300× *g* for 10 min and all the supernatant was discarded. The remaining white cell pellet was then resuspended in 10 mL of sterile deionized water and incubated for 30–40 min at 37 °C to complete the cell lysis. The lysate was centrifuged at 750× *g* for 15 min at room temperature, then the bacteria-containing cell pellet was resuspended in 10 mL of TiKa-Kic™ decontamination buffer (TiKa Diagnostics Ltd., London, UK) and the suspension was incubated overnight at 37 °C with shaking (150 rpm). After incubation, the suspension was centrifuged at 750× *g* for 15 min at room temperature and the pellet was resuspended in 1 mL of Difco™ Middlebrook 7H9 broth (Becton Dickinson—BD, Europe) and supplemented with 10% OADC enrichment, 2 μg/mL of Mycobactin J (IDVet, Grabels, France), and 1 μL of each TiKa™ Supplement kit (A, M1, M2, and M3; TiKa Diagnostics Ltd., London, UK) to allow the mycobacteria [27]. The supplemented suspension was then transferred to a 2 mL screw cap microtube and further incubated at 37 °C for 2 days, without shaking. After incubation, the suspension was transferred to a 4 mL BBL™ MGIT Mycobacteria Growth Indicator Tube (BD) and supplemented with 2 μg/mL Mycobactin J, 10% OADC enrichment, 100 μL of BBL™ MGIT™ PANTA™ (BD), and 5.1 μL of each TiKa Supplement 3. The supplemented MGIT tubes were then incubated at 37 °C and screened monthly for mycobacterial detection using an auramine–rhodamine fluorescence kit (TB-Fluor Phenol-Free set, Merck, Sigma-Aldrich Portugal), according to the manufacturer’s instructions. In presumptive mycobacteria-positive tubes (acid-fast bacilli (AFB)-positive), the MAP identity was confirmed by an IS900-based and F57-based real-time (RT)-PCR as previously described [29], on PBS-washed 1 mL aliquots taken from the base of the tube. The MGIT cultures were kept incubated at 37 °C until the MAP positivity was detected or at a maximum period of 18 months. If MAP positivity was detected, the cultures were kept thereafter at room temperature.

### 2.3. Faecal Cultures

For the *E. coli* culture, 0.5 g of faeces from each participant were suspended in 2 mL of sterile saline and vigorously shaken to homogenize it. The resulting suspension was then diluted 1:10^5^ in saline and 100 μL were used to inoculate a plate of CHROMagar™ Orientation medium (CHROMagar™ Microbiology, Paris, France), supplemented with 4 mg/L of vancomycin. The plates were incubated at 37 °C for 24 h. If no growth was observed, new plates were inoculated from more concentrated dilutions, that were kept at 4 °C. Purple *E. coli*-presumptive colonies, representative of each colony morphotype, were collected and sub-cultured on a new vancomycin-supplemented CHROMagar™ Orientation plate. The plates were incubated at 37 °C for 24 h and a second sub-culture was performed on a new plate without vancomycin. After another incubation period of 24 h at 37 °C, a collective suspension from all colonies of the same morphotype was prepared in 1 mL of Tryptic Soy Broth (TSB) in a microtube; this suspension was frozen at −70 °C. An aliquot of each culture was tested for *E. coli*-identity confirmation by a *mal*B-based RT-PCR, as described below.

For the MAP culture, 1 g of faeces from each patient was suspended in 5 mL of sterile PBS and vigorously shaken to homogenize it. The suspension was filtered through a 40 μm cell strainer and the filtrate was centrifuged at 1200× *g* for 30 min at room temperature. The supernatant was discarded, the pellet was then resuspended in 5 mL of PBS, and 150 μL of this suspension were added to 10 mL of the TiKa-Kic decontamination buffer. The resulting suspension was incubated at 37 °C for 24 h at 150 rpm. After incubation, the tube was centrifuged at 1200× *g* for 30 min. The supernatant was discarded, and the remaining pellet was resuspended in 0.5 mL of PBS. This total volume was used to inoculate a 4 mL MGIT tube supplemented with 2 μg/mL of Mycobactin J, 10% OADC enrichment, 100 μL of MGIT PANTA and 5.1 μL of TiKa Supplement A. The supplemented MGIT tubes were then incubated at 37 °C and screened monthly for mycobacterial detection, as described for the MAP blood cultures.

### 2.4. Confirmation of E. coli and MAP Identity Using Real-Time PCR

DNA was extracted from 100 μL of each *E. coli* suspension in TSB using the Quick-DNA™ Fungal/Bacterial Miniprep Kit (Zymo Research, Irvine, CA, USA), according to the manufacturer’s instructions. Confirmation of *E. coli* DNA was carried out by RT-PCR, using the primers ECO-1 (GACCTCGGTTTAGTTCACAGA) and ECO-2 (CACACGCTGACGCTGACCA) [30]. These primers amplify a region in the promoter of the *mal*B gene, resulting in a 585 bp amplification product. The PCR reaction was performed in a final volume of 20 μL, using the following reaction conditions: 10 μL SsoAdvanced Universal inhibitor-tolerant SYBR Green Supermix (Bio-Rad, Hercules, CA, USA), 0.5 μL of primer ECO-1 (0.5 μM final concentration), 0.5 μL of primer ECO-2 (0.25 μM final concentration) and 9 μL of template DNA. The cycling conditions were one cycle of 95 °C for 3 min, 40 cycles of 95 °C for 15 s, 59 °C for 15 s and 72 °C for 40 s, one cycle at 95 °C for 15 s, 60 °C for 15 s and then 96 °C, followed by one cycle at 30 °C for 30 s.

For the MAP DNA extraction, 300 μL were aspirated from the bottom of each presumptive mycobacteria-positive MGIT culture and were centrifuged at 11,000× *g* for 10 min. The supernatant was discarded, and the pellet was resuspended in 600 μL of Mycobacterial Lysis Buffer (MLB) [31] and incubated overnight at 37 °C with shaking. After incubation, the total volume was transferred to a ZR BashingBead™ Lysis Tube (included in the Quick-DNA™ Fungal/Bacterial Miniprep Kit, Zymo Research, Irvine, CA, USA) and the DNA was extracted following the manufacturer’s instructions. Confirmation of MAP DNA was carried out by an IS900- and F57-based RT-PCR, as previously described [29]. Only cultures with a positive amplification of both PCR products were confirmed as positive.

### 2.5. Investigation of AIEC Identity

*E. coli* isolates were assessed phenotypically for investigation of the AIEC identity, as described in [16,32], with some modifications. The Caco-2 cell line was used for the adhesion and invasion assays, while the murine J774 cell line was used to assess the survival and replication inside the macrophages. These cell lines were provided by the “Tumour and Microenvironment Interactions” research group, from the Instituto de Investigação e Inovação em Saúde (i3S, Porto, Portugal). All assays were performed in triplicate and all incubations were carried out at 37 °C and in a 5% CO_2_ atmosphere. The Caco-2 cells were plated in Basal Medium Eagle (Sigma-Aldrich) supplemented with 20% FBS (GIBCO), 1 mM sodium pyruvate (GIBCO) and 0.1 mM non-essential amino acids (GIBCO). The cultures were established in 24-well plates at a density of 4 × 10^5^ cells/well and incubated for 20 h. The cells were infected with the *E. coli* isolate at a multiplicity of infection (MOI) of 10:1 and were incubated for 3 h. The cells were then washed with PBS to remove extracellular non-adherent bacteria. For the adhesion assays, 1% Triton X-100 was added to lyse the cells. The bacterial burden was determined in each well by plating serial dilutions into LB agar (NZYTech, Lisboa, Portugal) and performing CFU counts after a 24 h incubation period at 37 °C. The adhesion index (ADH-I) was determined by calculating the mean number of bacteria per cell. For the invasion assays, a fresh medium supplemented with 100 μg/mL of gentamicin (Sigma-Aldrich) was added after cell washing, and the cells were incubated for an additional hour to kill extracellular bacteria. Then, 1% Triton X-100 was added, and the bacterial burden was determined as described above. The invasion index was expressed as the percentage of the initial inoculum that became intracellular, calculated as INV-I (%) = (intracellular bacteria/4 × 10^6^ bacteria inoculated) × 100 [32]. The ability of bacteria to replicate inside the macrophages was assessed by seeding 2 × 10^5^ J774 cells per well in an RPMI 1640 supplemented with 10% FBS and 2 mM glutamine (Sigma-Aldrich Portugal). Cultures were established in duplicate plates and incubated for 20 h to obtain cell monolayers. Then, the medium was replaced, and the monolayers were infected at an MOI of 10:1. The plates were centrifuged at 900 rpm for 10 min and then incubated for another 10 min, washed with PBS and 100 μg/mL of gentamicin was added to each well. After a 40 min incubation period, one plate was washed with PBS, the cells were lysed and the number of CFU/mL was determined as described above. In the second plate the culture medium was replaced, 20 μg/mL of gentamicin was added per well and the plates were incubated for an additional 23 h period (24 h time point). Then, the cells were washed with PBS and lysed, and the bacterial numbers were determined as described. The replication index (REP-I) was expressed as the mean percentage of bacteria recovered at 1 h and 24 h post-infection: REP-I (%) = (bacteria at 24 h/bacteria at 1 h) × 100 [32]. The AIEC phenotype was confirmed when the ADH-I was equal to or higher than 1, the INV-I was equal to or higher than 0.1% and the REP-I was equal to or higher than 100% [32].

### 2.6. MAP Sub-Culture and Genome Sequencing

MGIT cultures confirmed as positive for MAP by RT-PCR were then sub-cultured in RAFO14D medium (with 38 mM D/L asparagine, 15 mM potassium dihydrogen phosphate, 8 mM magnesium sulphate, 8 mM tri-ammonium citrate, 1.6 mM sodium chloride, 1.1 mM D(+) glucose, 0.3 mM ammonium iron, 5% glycerol, 2 μg/mL mycobactin J, 10% OADC, and 1 μg/mL TiKa peptide 14D (1 mg/mL), 25 μg/mL amphotericin B, and 35 μg/mL nalidixic acid), for up to 12 months. The MAP genome sequencing was performed on three RAF sub-cultures showing colony growth and positive acid-fast staining (after 5—6 months). These sub-cultures were obtained from the blood of one cirrhotic patient, and from both blood and faecal cultures of the same CD patient. Briefly, the genomic DNA was extracted from PBS-washed cultures, stripped in methanol:choroform (2:1) for 10 min, and heated at 80 °C for 20 min. Prior to extraction, these cultures were additionally treated as follows: 800 μg/mL of lysozyme for 30 min at 37°C; 150 μg/mL of Proteinase K and 1% SDS for 30 min at 65 °C; 1% CTAB in 0.4 mM sodium chloride and TrisHCl/EDTA for 30 min at 65°C. The mixture was then extracted with 1:1 chloroform:isoamyl alcohol (24:1), precipitated overnight at −20 °C with *v*/*v* isopropanol, washed in 70% ethanol, and resuspended in TE. The DNA concentration and integrity were determined using a Qubit High Sensitivity DNA assay (Life Technologies, Thermo Fisher Scientific, UK) and Agilent Tapestation 2100 using a genomic screentape, respectively. Library preparation and sample indexing were undertaken using NexteraXT DNA, according to the manufacturer’s instructions, followed by bead-based normalization and pooling with other libraries. Sequencing was performed using MiSeq Reagent Kits v3, with 2 × 300 bp paired-end reads, in the Illumina MiSeq sequencing platform. The reads were mapped to the MAP reference genome (RefSeq accession: NC_002944.2) using bwa mem v0.7.3a-r367 [33]. The alignments were sorted, duplicates were removed with samtools v0.1.19 [34] and site statistics were generated using samtools mpileup. The compiled genome was mapped against other mycobacteria using the NCBI Sequence Viewer (Version 3.36.0, NCBI, Rockville, MD, USA) and Snapgene (Version 4.3.11, GSL Biotech, Boston, MA, USA).

## 3. Results

### 3.1. Characterization of the Cohort

This prospective study included 63 participants: 22 healthy controls (HC), 18 CD patients, 15 UC patients, and 7 cirrhotic patients (this group was included as a pathological control: patients with a digestive tract pathology other than IBD, with an impact on intestinal permeability) (Table 1).

Regarding the Montreal classification, most CD patients were diagnosed between the ages of 17 and 40 (A2—12 patients) and presented with a non-stricturing, non-penetrating disease behaviour (B1—10 patients), with involvement of the ileum (L1 or L3—14 patients). Most UC patients (9 out of 15 patients) had left-sided colitis (E2). Almost all the IBD patients were in clinical remission at the time of sample collection, with only two CD patients presenting mild activity according to the Harvey–Bradshaw index. Concerning the therapeutic regimen, most IBD patients were receiving biological therapy with adalimumab (ADA) or infliximab (IFX), either alone or in combination with azathioprine (AZA).

Regarding the cirrhosis group, all the patients had alcoholic liver cirrhosis and three had been abstinent for at least 6 months at the time of the sample collection (42.9%). Five (71.4%) patients had ascites. Only one patient with cirrhosis, who had previously undergone a liver transplant, was receiving immunosuppressive therapy with mycophenolate mofetil and tacrolimus.

### 3.2. E. coli Culture and AIEC Identity

Of the 63 haemocultures established in the Castañeda bottles, only two showed one *E. coli* presumptive colony in the solid phase (3.2%). Although the Castañeda medium was developed for the isolation of *Brucella*, other Gram-positive and Gram-negative bacteria may also be isolated with this system, including *E. coli,* and other members of the Enterobacteriaceae family [35,36]. These presumptive colonies, however, were sub-cultured into CHROMagar™ plates, but no growth was observed, and the flasks were discarded. In contrast, faecal *E. coli* isolates were obtained from most participants, with one or two *E. coli* presumptive colony morphotypes identified in CHROMagar™ cultures established from each faecal sample (Table 2).

*E. coli* identity was confirmed in 19/22 HC subjects, 4/7 patients with cirrhosis, 16/18 CD patients, and 14/15 UC patients, using a *mal*B promoter-based real-time PCR (Table 3).

UC patients (93.3%), CD patients (88.9%), and HC (86.4%) were the groups with higher frequency of confirmed faecal *E. coli* colonization, followed by cirrhotic patients (57.1%), although differences were not statistically significant (Table 3). Of all confirmed *E. coli* isolates, only three showed features suggestive of AIEC identity (isolates CD1, UC5A and UC13A), although not all AIEC criteria were met (Table 4).

### 3.3. MAP Culture

For MAP culture, we used conventional MGIT tube media enriched with TiKa supplements that are reported to increase MAP recovery from clinical samples [27]. Positive fluorescence in MGIT is usually a sign of mycobacterial growth; however, previous observations by our team have suggested that MAP metabolism is so slow that in many cases, the growth does not trigger a fluorescent signal.

We thus decided to screen our MGIT cultures monthly for acid fastness using an auramine–rhodamine-based detection commercial kit and then confirmed the MAP presence in suspected tubes using an IS900- and F57-based RT-PCR. Comparative frequencies of the MAP-positive cultures obtained from faecal and blood samples from each subject group are depicted in Table 5.

Although no significant differences in the MAP MGIT culturability from the faecal samples was observed between the subject groups, we observed a trend towards a higher MAP MGIT culturability in the CD patients (11.1%) followed by the UC patients (6.7%), while no MAP-positive confirmation was obtained in faecal MGIT cultures from the HC and cirrhosis groups.

Regarding the blood MGIT cultures, we observed a higher trend for MAP culturability in the cirrhosis patients (14.3%), followed by the CD patients (11.1%). MAP culturability in the MGIT medium was not confirmed in the other two subject groups (i.e., the HC and UC groups). MAP recovery in the RAFO14D sub-cultures established from IS900-positive MGIT cultures was only achieved in 3/7 strongly IS900-positive samples (Table 6).

No MAP recovery was possible from the weakly IS900-positive MGIT cultures. Sequencing of the sub-cultures established from the faeces and the blood of one CD patient (CD3) suggested the presence of the same clonal MAP strain in the two settings. This stain had a deletion in genes associated with a ferredoxin (results not shown). The other strain sequence was obtained from a blood sample of a patient with liver cirrhosis and was very close to the type strain MAP4, which has been isolated from human breast milk and is annotated in the Genbank as MAP4_RS15540.

## 4. Discussion

The identification of pathobionts with a role in IBD is of importance to understand the complex multifactorial process of this disease [37,38,39]. Here, we studied the colonization of our patient cohort by viable AIEC and MAP.

Although we were not able to detect *E. coli* growth from blood samples, the majority of the faecal samples were *E. coli* colony-positive in CHROMagar^TM^. Only a small number of presumptive *E. coli* colonies, as determined by colony observation on a CHROMagar™ orientation medium, were not confirmed as *E. coli*-positive using the *mal*B promoter-based real-time PCR (results not shown). CHROMagar™ was developed for urinalysis and, according to the manufacturer, has a specificity of 99.3% for *E. coli* isolated from urine samples (CHROMagar™ product leaflet). A lower specificity may occur with other sample types, although a similarly high specificity of *E. coli* detection was reported for different sample sources. It is, nevertheless, possible that due to the high intestinal microbial burden of other microbial species, it may produce colonies identical in colour to *E. coli*, thus resulting in false positives.

Despite the considerable number of studies pointing to an association between colonization by AIEC variants and CD [9,14], our study did not confirm this. Indeed, MAP was more frequently recovered from the blood samples of CD patients than *E. coli*. The lack of *E. coli* in our cohort’s haemocultures may reflect the lack of access of the enteropathogens to the bloodstream (as was most likely the case in subjects with a functional intestinal barrier), or the rapid clearance of circulating bacteria by the immune system, even in the presence of intestinal leakage. Concerning faeces, among the 53 *E. coli* isolates, only three (with one from a CD patient and two from individuals with UC) were compatible with an AIEC-like phenotype. This low number may be related to the use of faecal samples instead of ileal biopsies, since AIEC may be more diluted in faeces and be difficult to detect. Not all criteria of AIEC identity were met by our samples, according to [32]. Indeed, CD1 and UC5A isolates showed, respectively, REP-I or ADH-I values below those established for AIEC identity. Moreover, REP-I was not possible to be obtained for the UC13A isolate, since it was gentamicin-resistant. As our reference *E. coli* strain (ATCC 25922) showed an ADH-I of 0.86 and an INV-I of 0 (zero), however, we assumed that our three IBD isolates had AIEC-like features. Previous studies have identified *E. coli* isolates from CD with mixed features of AIEC and other *E. coli* variants [40]. Recently, it was demonstrated that AIEC comprises a spectrum of isolates that varies in its adhesion/invasion/replication capacity, with the isolates not matching all the criteria established for the reference AIEC variants [41]. Nonetheless, our results do not confirm an association between AIEC colonization and IBD as previously suggested by [11,42]. Further investigation of the genetic diversity of the *E. coli* confirmed isolates will be important to identify possible new *E. coli* variants.

Intracellular MAP (i.e., a proven animal inflammatory pathogen) has been reported to trigger pro-inflammatory responses. Its presence has been associated with a range of human and animal inflammatory diseases [43,44,45]. Additionally, MAP-specific antibodies have been detected in CD patients [46]. Difficulties in patient enrolment (due to the implementation of COVID-19 restrictions) prevented us from achieving statistically significant differences in our cohorts. Nevertheless, MAP was detected in the MGIT cultures from both faecal and blood samples of CD patients, which supports an association between MAP and CD. No association was found between MAP and AIEC positivity. Indeed, the MAP-positive samples from the CD patients were negative for AIEC and the single CD patient with a faecal AIEC isolate was MAP-negative. Interestingly, the genome sequences from the faecal and the blood samples of the same CD patient were indicative of infection/persistence of the same strain in the gut mucosa/lumen and in the blood. This suggests that the route of MAP entry to the bloodstream is the gut mucosa and is likely to result from a chronic persistent passage between the two compartments.

From our cohort of 18 CD patients (Table 1), 16 were in clinical remission and only 2 (i.e., CD5—MAP-negative blood culture and CD10—MAP-positive blood culture) had mildly active disease. No UC patients with active disease were included. Hence, MAP positivity in the blood cultures of IBD patients in remission (with the majority under infliximab treatment, which has been associated with a restoration of the intestinal barrier function), strongly suggests that MAP was not transiently infecting these patients, but, instead, should have been present for some time [47]. Entry possibly occurred through the intestinal mucosa in a stage of active disease. Once this microorganism enters and infects cells in the blood, such as peripheral blood leukocytes, there is no evidence that MAP can be eliminated [25]. It is possible, therefore, that infection is not ever dependent upon consistent permeability but is more about the inability of the infected monocytes to eliminate the pathobiont.

Despite MAP positivity in the MGIT cultures, recovery of ‘culturable’ MAP (MGIT) in RAFO14D sub-cultures was only achieved in three out of seven strongly IS900-positive cultures, and zero out of eight weakly IS900-positive cultures. Two of these were from the same patient and all three required long-term incubation in specialized peptide-stimulating media. This suggests that MAP, once taken up intracellularly in human blood cells, develops a strongly viable and non-culturable phenotype. The use of next-generation sequencing (NGS) may constitute an alternative to MAP culture in detecting the presence of viable organisms through metatranscriptomics [48]. The NGS approach, however, would require samples with much higher MAP numbers and with low background (due to other organisms or host targets); therefore, we decided to use a culture-based approach. Further research is needed to improve media that will more effectively convert these phenotypes and make isolation easier and more time-effective.

In conclusion, our study shows that viable MAP persists readily within human blood leukocytes in both patients with cirrhosis and CD. Although extensively described in the literature, we did not find a strong association between the presence of AIEC and CD. The long-term persistence of intracellular MAP in the bloodstream may lead to chronic inflammatory exacerbation and play a role in disease reactivation. Its etiologic role in CD, however, is still unproven. Further studies on larger cohorts, including patients with active disease, with more severe phenotypes, and under other therapies, are needed to clarify whether the persistence of these pathobionts is a cause or a consequence of IBD and whether it relates to disease activity and prognosis.

## Figures and Tables

**Table 1:** Subjects enrolled in the study.

		HC	CD	UC	CIR
Number (*n*)	-	22	18	15	7
Age	Mean	66.0	34.4	46.5	67.0
	Range	14–84	18–65	18–72	57–83
Gender (%)	Male	15	10	10	7
	Female	6	8	5	0
Disease duration (years)	Mean	—	10.4	11.0	4.4
	Range	—	1–24	3–20	0.2–14
Montreal classification (CD)	A1	—	0	—	—
	A2	—	12	—	—
	A3	—	3	—	—
	L1	—	6	—	—
	L2	—	4	—	—
	L3	—	3	—	—
	B1	—	10	—	—
	B2	—	2	—	—
	B3	—	4	—	—
Montreal classification (UC)	E1	—	—	2	—
	E2	—	—	9	—
	E3	—	—	4	—
Harvey–Bradshaw (CD)	<5 (remission)	—	16	—	—
	5–7 (mild)	—	2	—	—
Mayo (UC)	0–1 (remission)	—	—	15	—
Child–Pugh classification (cirrhosis)	A	—	—	—	3
	B	—	—	—	3
	C	—	—	—	1
Therapy	ADA/IFX	—	10	7	—
	ADA/IFX + AZA	—	5	2	—
	ADA/IFX + 5-ASA	—	1	2	—
	AZA	—	2	0	—
	ADA/IFX + AZA + 5-ASA	—	0	4	—

HC: Healthy controls; CD: Crohn’s disease; UC: Ulcerative colitis patients; CIR: cirrhotic patients; ADA: Adalimumab; IFX: Infliximab; AZA: azathioprine; 5-ASA: 5-aminosalicylic acid.

**Table 2:** Presumptive *E. coli* isolates obtained from faecal samples.

	No Presumptive *E. coli* Isolates	One Presumptive *E. coli* Isolate	Two Presumptive *E. coli* Isolates (A and B)
HC	HC3, HC4, HC19	HC7, HC8, HC10, HC12, HC13, HC14, HC16, HC18, HC21	HC1, HC2, HC5, HC6, HC9, HC11, HC15, HC17, HC20, HC22
CIR	CIR1, CIR4, CIR7	CIR2, CIR3, CIR5	CIR6
CD	CD5, CD7	CD1, CD2, CD6, CD9, CD10, CD13, CD14, CD15, CD17, CD18, CD19	CD3, CD4, CD8, CD12, CD16
UC	UC3	UC2, UC4, UC6, UC7, UC11, UC15, UC16, UC17	UC1, UC5, UC8, UC12, UC13, UC14

HC Healthy controls; CD: Crohn’s disease patients; UC: Ulcerative colitis patients; CIR: Patients with cirrhosis. Purple colonies of diverse morphotypes on CHROMagar™ Orientation R agar medium were considered as presumptive *E. coli* isolates.

**Table 3:** Frequency of subjects with confirmed *E. coli*-positive samples.

	Confirmed *E. coli-*Positive Samples—*n* (%) *	
	FAECAL SAMPLES	BLOOD SAMPLES
Healthy controls	19 (86.4%)	0 (0.0%)
Patients with cirrhosis	4 (57.1%)	0 (0.0%)
Crohn’s disease patients	16 (88.9%)	0 (0.0%)
Ulcerative colitis patients	14 (93.3%)	0 (0.0%)

* *mal*B promoter-based real-time PCR testing of each EC-suspected culture morphotype on CHROMagar™ Orientation medium.

**Table 4:** Putative AIEC identity of *E. coli* isolates obtained from faecal cultures.

EC Isolate	ADH-I	INV-I	REP-I
CD1	1.81	0.11	64.12
UC5C	0.9	0.17	188.24
UC13C	2.19	ND	ND

ADH-I: Adhesion Index; INV-I: Invasion Index; REP-I: Replication Index. Indexes calculated as described in Materials and Methods section. An EC isolate is considered AIEC when ADH-I > 1, INV-I > 0.1 and REP-I > 100. ND: not detected, due to gentamycin resistance of the isolate.

**Table 5:** Frequency of subjects with confirmed MAP-positive samples, and positive sub-culture in RAF014D medium (within the confirmed MAP-positive sample).

	FAECAL SAMPLES		BLOOD SAMPLES	
	MAP-Positive— *n* (%) *	Positive Sub-Culture in RAF014D	MAP-Positive— *n* (%) *	Positive Sub-Culture in RAF014D
Healthy controls	0 (0.0%)	0 (0.0%)	0 (0.0%)	ND
Patients with cirrhosis	0 (0.0%)	0 (0.0%)	1 (14.3%)	1 (100.0%)
Crohn’s disease patients	2 (11.1%)	1 (50.0%)	2 (11.1%)	1 (50.0%)
Ulcerative colitis patients	1 (6.7%)	0 (0.0%)	0 (0.0%)	0 (0.0%)

* IS900- and F57-based real-time PCR testing of each MAP-suspected culture (detected as acid-fast acilli-positive).

**Table 6:** MAP RT-PCR and sub-culture results on samples positive for IS900-based RT-PCR. A weak positive corresponded to a cycle threshold above 38.

			IS900 PCR	F57 PCR	Sub-culture in RAFO14D	AFB Stain on Sub-culture	Sequenced
**HC**	**Faeces**	HC1	Weak positive	Negative	NVG (12 months)	No AFB visible	No
**CIR**	**Blood**	CIR2	Weak positive	Negative	NVG (12 months)	No AFB visible	No
		CIR3	Weak positive	Negative	NVG (12 months)	No AFB visible	No
		CIR4	**Positive**	**Positive**	**Positive growth (8 months)**	**AFB seen**	**Yes**
		CIR6	Low positive	Negative	Scanty growth	Suspected AFB	No
		CIR7	Weak positive	Negative	NVG (12 months)	ND	No
**CD**	**Faeces**	CD3	**Positive**	**Positive**	**Positive growth (5 months)**	**AFB seen**	**Yes**
		CD9	**Positive**	Negative	NVG (12 months)	Suspected AFB	No
		CD12	Weak positive	Negative	NVG (12 months)	No AFB visible	No
		CD17	**Positive**	**Positive**	Scanty growth	Suspected AFB	No
	**Blood**	CD3	**Positive**	**Positive**	**Positive growth (6 months)**	**AFB seen**	**Yes**
		CD6	Weak positive	Negative	NVG (12 months)	No AFB visible	No
		CD10	**Positive**	Weak positive	Scanty growth	No AFB visible	No
		CD13	Weak positive	Negative	NVG (12 months)	No AFB visible	No
**UC**	**Faeces**	UC13	**Positive**	Weak positive	NVG (12 months)	No AFB visible	No

HC: Healthy controls; CIR: Cirrhotic patients; CD: Crohn’s disease patients; UC: Ulcerative colitis patients; NVG: no visible growth.

## Data Availability

Data may be provided at a reasonable request to the corresponding author.
